# Characteristics of Hospitalized Cases of Pertussis in Catalonia and Navarra, Two Regions in the North of Spain

**DOI:** 10.1371/journal.pone.0139993

**Published:** 2015-10-06

**Authors:** Inma Crespo, Diana Toledo, Núria Soldevila, Iolanda Jordán, Rubén Solano, Jesús Castilla, Joan A. Caylà, Pere Godoy, Carmen Muñoz-Almagro, Ángela Domínguez, Working Group on the Transmission of Pertussis in Households

**Affiliations:** 1 CIBER Epidemiology and Public Health. Barcelona, Spain; 2 Public Health Department, University of Barcelona, Barcelona, Spain; 3 Public Health Agency, Barcelona, Spain; 4 Paediatric Intensive Care Medicine, Hospital Sant Joan de Déu, Barcelona, Spain; 5 Public Health Institute, Navarra Institute for Health Research, Pamplona, Spain; 6 Agency of Public Health of Catalonia, Barcelona, Spain; 7 Molecular Microbiology Department, Hospital Sant Joan de Déu, Barcelona, Spain; Universidad Nacional de la Plata, ARGENTINA

## Abstract

Pertussis causes a large number of cases and hospitalizations in Catalonia and Navarra. We made a study of household cases of pertussis during 2012 and 2013 in order to identify risk factors for hospitalization in pertussis cases. Each primary case reported triggered the study of their contacts. Close contacts at home and people who were in contact for >2 hours during the transmission period of cases were included. The adjusted OR and 95% confidence intervals (CI) was calculated using logistic regression. A total of 1124 pertussis cases were detected, of which 14.9% were hospitalized. Inspiratory whoop (aOR: 1.64; CI: 1.02–2.65), apnoea (aOR: 2.47; CI: 1.51–4.03) and cyanosis (aOR: 15.51; CI: 1.87–128.09) were more common in hospitalized than in outpatient cases. Hospitalization occurred in 8.7% of correctly-vaccinated cases, 41.1% of non-vaccinated cases and 9.4% of partially-vaccinated cases. In conclusion, inspiratory whoop, apnoea and cyanosis were associated factors to hospitalization while vaccination reduced hospitalizations due to pertussis.

## Introduction

Pertussis disease causes a high number of cases and hospitalizations even though it is a preventable disease with high vaccination coverages [1–3]. The vaccination schedule of pertussis in Spain includes the first series of vaccine at 2, 4, and 6 months and two booster doses at 18 months and 4–6 years [4,5]. Vaccination coverages in Spain were > 95% for the first series of vaccination, up to 90% for the first booster dose, and around 80% for the second booster dose in 2011 [6, 7]. In Spain, notification of pertussis disease is mandatory and must be reported by physicians to the Health Department. In recent years, the incidence of pertussis has increased in Catalonia and Navarra, in other regions in Spain, and worldwide [8], and an increase in hospitalizations has been observed. Reported reasons for the increasing incidence include improvements in diagnostic methods [9–10], the decrease in the protection provided by pertussis vaccination over time, and the fact that vaccination, or having the disease, does not provide life-long protection against new infection. Additional suggested reasons include waning immunity [11], the change from the whole cell to the acellular vaccine, vaccine failure, and changes in B. pertussis strains [12–14].

However, some reports suggest that vaccination with at least one dose reduces the risk of hospitalization [15–16]. Determination of risk factors for hospitalization could aid vaccination and decrease hospitalization due to pertussis and therefore reduce the disease burden for the public health system.

The aim of this study was to determine risk factors for hospitalization in cases of pertussis in two Spanish regions (Catalonia and Navarra).

## Methods

Between January 2012 and December 2013, all confirmed pertussis cases and their contacts were reported to the surveillance system in Catalonia and Navarra, which have a population of 8,200,000 (17.4% of the Spanish population). Pertussis cases were defined as patients with ≥ 2 weeks of cough with other symptom such as paroxysms, inspiratory whoop, posttussive vomiting or apnoea, accompanied by one of the following: laboratory confirmation by real-time polymerase chain reaction (PCR) or culture or epidemiologic link with a laboratory-confirmed case. Bacterial culture were prepared with selective charcoal agar (5%) plates (Oxoid, Basingstoke, UK), defibrinated human blood (10%) and cephalexin (40mg/l) (Selective supplement *Bordetella*, Oxoid, Basingstoke, UK). The plates were incubated in a humid atmosphere at 37°C (±2°C) and were examined daily for 5 days. The real-time PCR target used in the study was insertion sequence IS481 of *B*. *pertussis*. This sequence presents a high copy number in *B*. *pertussis* (about 200 copies) and a low copy number in other *Bordetella* species as *B*. *holmesii* or *B*. *bronchiseptica*. A second real-time PCR was performed in samples with Ct values above 35, and these samples were also concentrated (x4) by easyMAG (BioMerieux Laboratories, Marcy l’Etoile, France). The target insertion sequence was IS1002, which allow discrimination between other *Bordetella* spp and *B*. *pertussis*, as low copy numbers are found in *B*. *pertussis*.

A modified definition was used for patients aged < 1 year, in whom coughing could last < 2 weeks, but had to be accompanied by paroxysm and another symptom. Household contacts were defined as usual residents of the house of the index case; other contacts were defined as people who spent > 2 hours in the house of the index case during the transmission period of the disease.

Each contact was surveyed about exposure to the case, respiratory symptoms, doses of vaccine received and chemoprophylaxis. Once the index case was detected, we identified all household and other contacts, who were surveyed immediately after identification. In addition, both cases and contacts were followed for 28 days after the date of report in order to determine complications and the appearance of secondary cases.

Contacts with cough or other symptom related to pertussis were laboratory tested to confirm or rule out the disease. Some contacts became cases after laboratory testing and were included as cases, not as contacts. Pertussis index cases were defined as the first case reported and diagnosed that generated the household study. After the household study, index cases were classified as primary case, co-primary case or secondary case. Primary cases were cases without an epidemiological link to a pertussis case previously detected in the house. Co-primary cases were cases epidemiologically linked to a pertussis case diagnosed in the household with onset of symptoms between 0 and 6 days after the other pertussis case. Secondary cases were cases with an epidemiological link with a pertussis case previously detected in the household with onset of symptoms between 7 and 28 days after the other pertussis case. Symptoms from cases were obtained from clinical reports, from the attending physicians and by interview with cases or their parents or tutors. The following variables were collected in pertussis cases: clinical features, age, length of hospitalization, laboratory test used, vaccination status, date of onset of symptoms, date of diagnosis, vaccination status and type of case (primary or secondary). The following variables were collected in contacts: exposure to the case, respiratory symptoms, doses of vaccine received and chemoprophylaxis.

Diagnostic delay was calculated as the days between the onset of symptoms and the day of diagnosis. Cases were classified according to the vaccination status as follows: Correctly-vaccinated cases were cases that had received the number of doses corresponding to their age (and had received at least one dose). Non-vaccinated cases were cases which had not received any dose of vaccine. Partially-vaccinated cases were cases which had received some but not all vaccinations corresponding to their age. The Chi square test was used to compare the vaccination status.

The odds ratio (OR) and 95% confidence intervals (CI) between hospitalized and outpatient cases were calculated to determine the variables influencing the risk of hospitalization. To control for possible confounding variables, the adjusted OR was calculated using logistic regression, including other variables, such as symptoms and vaccination status, in the model. We also stratified the OR by age.

Population figures were taken from the National Institute of Statistics (INE) [17]. The statistical analysis was performed using the SPSS v.18 package. Statistical significance was established as α = 0.05.

The study was conducted according to the principles expressed in the Declaration of Helsinki. All cases included in the study received detailed information about the aims of the study. We first obtained verbal consent to participate and then written consent from all cases. When cases were minors, consent was obtained from relatives, carers or guardian. The study was approved by the Ethics Committee of Hospital Sant Joan de Deu in Barcelona, Spain.

## Results

In the study period, 668 pertussis cases were reported to surveillance units and included in the study. These generated 2793 household contacts, 456 of whom became pertussis cases and generated 59 new household contacts. Therefore, 1124 pertussis cases occurred during the study, of which 536 (47.7%) were male and 588 (52.3%) female.

Seven hundred and thirty-two cases were determined by PCR, 5 by culture, 32 by both PCR and culture, and 355 by epidemiological link. Seven hundred and thirty eight (65.7%) cases were classified as primary or co-primary and 386 (34.3%) as secondary.

Table 1 shows the median age, duration of cough and diagnostic delay in hospitalized and outpatient cases.

**Table 1:** Age, duration of cough and diagnostic delay in pertussis cases.

Cases	Hospitalized (n = 167)median (range)	Outpatient (n = 936)median (range)	p value
**Age (years)**	**0 (0–50)**	**10 (0–87)**	**<0.001**
**Duration of cough (days)**	**30 (4–98)**	**30 (3–112)**	**0.326**
**Diagnostic delay (days)**	**10 (1–67)**	**18 (0–136)**	**<0.001**

One hundred and sixty-seven (14.9%) cases were hospitalized, 936 (83.3%) were outpatient cases and no information was available in 21 cases. The hospitalization rate was 1.01 per 100,000 inhabitants. Eighty-four hospitalized cases (50.3%) were male and 83 (49.7%) female (p = 0.511). Eighty-two (49.1%) cases were hospitalized in 2012 and 85 (50.9%) in 2013 (p = 0.221).

Of the 167 hospitalized cases, 163 (97.6%) were index cases, of which 100 (61.3%) were primary or co-primary cases and 63 secondary cases. Of the 4 cases that were not index cases, 3 (75.0%) were primary cases and 1 was a secondary case. Secondary hospitalized cases acquired the infection most frequently from the mother (26.5%), siblings (23.4%), the father (20.3%), other relatives (20.3%) and grandparents (9.5%).

One hundred and fifty four hospitalized cases (92.2%) were confirmed by PCR, 8 (4.8%) by both PCR and a positive culture test, 1 (0.6%) by a positive culture and 4 cases (2.4%) were epidemiologically-linked to a previous laboratory-confirmed case.

**Fig 1:**
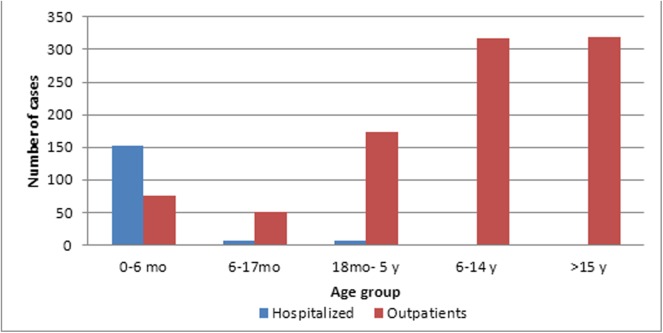
Number of hospitalized and outpatient pertussis cases according to age group. mo. months; y. years.

Differences in age group between outpatient and hospitalized cases are shown in Fig 1. Of hospitalized cases, 91% were aged < 6 months, while 34% of outpatient cases were aged > 15 years.

Table 2 shows the symptoms in hospitalized and outpatient cases. Cough ≥ 2 weeks was excluded from the table, as it was mandatory in reporting a case. The adjusted OR for symptoms shows that inspiratory whoop, apnoea and cyanosis were associated with hospitalization. In pertussis cases aged 0 to 6 months, apnoea was associated with hospitalization (S1 Table).

**Table 2:** Predictive symptoms in hospitalized and outpatient pertussis cases.

	Hospitalized n = 167	Outpatient n = 936	raw OR (95%CI)	p value	adjusted ORa (95%CI)	p value
**Paroxysmal cough**	153	771	2.66 (1.44–4.91)	0.002	2.21 (0.91–5.37)	0.081
**Inspiratory whoop**	94	303	2.82 (2.01–3.97)	<0.001	1.64 (1.02–2.65)	0.040
**Posttussive vomiting**	93	332	2.40 (1.71–3.37)	<0.001	1.04 (0.64–1.67)	0.869
**Apnoea**	89	160	5.66 (3.98–8.05)	<0.001	2.47 (1.51–4.03)	<0.001
**Fever**	25	77	1.96 (1.20–3.18)	0.007	1.26 (0.63–2.52)	0.514
**Cyanosis**	12	1	74.46 (9.61–576.98)	<0.001	15.51 (1.87–128.09)	0.011
**Pneumonia**	5	6	4.81 (1.45–15.95)	0.010	33.58 (0.17–6395.84)	0.189

a. Adjusted for the following variables: Symptoms, Age and Vaccination status.

The vaccination status was known in 905 (81.5%) cases. Table 3 shows the vaccination status in hospitalized and outpatient cases.

**Table 3:** Vaccination status in hospitalized and outpatient cases.

Cases	Hospitalized (n = 167) n (%)	Outpatient (n = 738) n (%)
**Correctly vaccinated (n = 539)**	47 (8.7)	492 (91.3)
**Partially vaccinated (n = 96)**	9 (9.4)	87 (90.6)
**Non-vaccinated (n = 270)**	111 (41.1)	159 (58.9)

There were fewer hospitalizations in correctly-vaccinated cases than in non-vaccinated cases (p<0.001), and fewer hospitalizations in partially-vaccinated cases than in non-vaccinated cases (p<0.001). Of hospitalized cases, 56 had received some dose of vaccine: 41 had received 1 dose (73.2%), 7 cases 2 doses (12.5%), 3 cases 3 doses (5.3%), 3 cases 4 doses (5.3%) and 2 cases had received 5 doses (3.7%).

## Discussion

A large number of pertussis cases were detected in 2012 and 2013 in Catalonia and Navarra, of which approximately 15% were hospitalized (> 90% were aged <1 year). More than half the cases detected were primary or co-primary cases, an important factor for both early treatment and breaking the transmission of the disease.

Our results show that some symptoms were more frequent in hospitalized than in outpatient cases, including inspiratory whoop, apnoea and cyanosis (which is not a symptom but a complication of the disease). A New Zealand study found that paroxysmal cough and cyanosis were predictive factors for not discharging a case from hospital, in order to avoid readmission [18]. A Swiss study found that hospitalized cases more frequently had cyanosis [19].

We found a reduction in hospitalizations when patients were correctly vaccinated or partially vaccinated. This reinforces the importance of vaccination and suggests that vaccination reduces disease severity and protects against hospitalization. Similar conclusions have been reported by other authors, and in some studies one dose of vaccine was sufficient to produce this reduction [15–16, 19–21].

Some studies have reported that whole cell pertussis vaccine offers greater protection than the acellular vaccine. We could not analyse this factor, as all hospitalized cases in our study were aged <10 years (except 1 case aged <45 years with unknown vaccination status) and all were vaccinated with the acellular vaccine, which has been available in Catalonia since 2002 [22].

Previous studies in Catalonia found a large percentage of pertussis cases in infants [23–25]. In our study, which included active surveillance, a large number of cases in patients aged 15–44 years were detected. Normally a diagnosis of pertussis is not made in this age group as they have less-severe disease than infants [26–27]. However, several studies in different countries have found that the age pattern of pertussis has changed and the incidence of cases in adolescents and adults has increased [28–33]. This may be because the vaccination coverage in older people is lower than in infants and also because of a loss of immunity [34].

Several studies have found that unreported and misdiagnosed cases are a public health problem [35–36]. Our study avoided this problem because we made active surveillance. For this reason, we found a higher incidence in adults in Catalonia and Navarra than previously described [6, 8].

A limitation of the study is the large number of epidemiologists involved. Although all epidemiologists applied the same protocol, we cannot rule out differences in the interpretation of some variables. However, we believe this did not affect the results, as the Working Group made a considerable effort to correct possible differences in data interpretation.

Regarding PCR diagnosis, *B*. *bronchiseptica* strains that contain a copy of IS481 (it is estimated that IS481 is present in only 1% of *B*. *bronchiseptica*) could by erroneously identified as *B*. *pertussis* [37].

In conclusion, we found that inspiratory whoop, apnoea and cyanosis were good predictors of hospitalization in pertussis cases. Vaccination reduces the disease severity and hospitalizations.

## Supporting Information

S1 Table Predictive symptoms in hospitalized and outpatient pertussis cases by age group. (DOCX)
