# Supplementary material for: Characteristics of Hospitalized Cases of Pertussis in Catalonia and Navarra, Two Regions in the North of Spain
Source: PLoS One. 2015 Oct 6;10(10):e0139993. doi: 10.1371/journal.pone.0139993 (PMC4595087; doi:10.1371/journal.pone.0139993)
Supplement: S1 Table — (DOCX) [file pone.0139993.s001.docx]

Supporting Information

S1 Table. Predictive symptoms in hospitalized and outpatient pertussis cases by age group.

| **Symptoms** | **Hospitalized**  **(%)** | **Outpatient**  **(%)** | **OR raw**  **(95%CI)** | **p value** | **OR adjusted^1^**  **(95%CI)** | **p value** |
| --- | --- | --- | --- | --- | --- | --- |
| **0-6 months (n=228)** | | | | | | |
| **Paroxysmal cough** | 140 (92.7) | 63 (84) | 2.42  (1.01-5.78) | 0.046 | 2.19  (0.72-6.61) | 0.163^a^ |
| **Inspiratory whoop** | 86 (57.7) | 32 (43.2) | 1.79  (1.02-3.14) | 0.042 | 1.46  (0.76-2.81) | 0.252^b^ |
| **Posttussive vomiting** | 83 (56.1) | 39 (52) | 1.17  (0.67-2.05) | 0.563 | 0.87  (0.45-1.69) | 0.695^c^ |
| **Apnoea** | 80 (53.7) | 21 (28) | 2.98  (1.63-5.42) | <0.001 | 2.47  (1.25-4.87) | 0.009^d^ |
| **6-17 months (n=58)** | | | | | | |
| **Paroxysmal cough** | 4 (80) | 45 (88.2) | 0.53  (0.51-5.59) | 0.601 | 0.25  (0.10-6.31) | 0.401^e^ |
| **Inspiratory whoop** | 3 (60) | 21 (41.2) | 2.14  (0.32-13.96) | 0.425 | 0.77  (0.06-8.86) | 0.841^f^ |
| **Posttussive vomiting** | 4 (66.7) | 29 (56.9) | 1.51  (0.25-9.04) | 0.647 | 2.51  (0.18-34.95) | 0.493^g^ |
| **Apnoea** | 5 (83.3) | 16 (32) | 10.62  (1.14-98.58) | 0.038 | 8.97  (0.57-139.15) | 0.117^h^ |
| **18 months - 14 years (n=507)** | | | | | | |
| **Paroxysmal cough** | 8 (100) | 404 (82.6) | - |  | - |  |
| **Inspiratory whoop** | 4 (57.1) | 158 (32.6) | 2.75  (0.61-12.44) | 0.189 | 2.04  (0.41-10.31) | 0.377^i^ |
| **Posttussive vomiting** | 5 (71.4) | 196 (40.4) | 3.68  (0.71-19.19) | 0.121 | 2.28  (0.42-12.31) | 0.338^j^ |
| **Apnoea** | 3 (37.5) | 66 (13.6) | 3.81  (0.88-16.31) | 0.072 | 1.41  (0.24-8.26) | 0.705^k^ |

1. Adjusted for the following variables: Symptoms, Age and Vaccination status; a. power=43.9%; b. power=53.8%; c. power=8.3%; d. power=96.4%; e. power=3.3%; f. power=11.9%; g. power=7.5%; h. power=78.5%; i. power=28.7%; j. power=37.1%; k. power=49.2%.
